# Radiative lifetime-encoded unicolour security tags using perovskite nanocrystals

**DOI:** 10.1038/s41467-021-21214-3

**Published:** 2021-02-12

**Authors:** Sergii Yakunin, Jana Chaaban, Bogdan M. Benin, Ihor Cherniukh, Caterina Bernasconi, Annelies Landuyt, Yevhen Shynkarenko, Sami Bolat, Christoph Hofer, Yaroslav E. Romanyuk, Stefano Cattaneo, Sergey I. Pokutnyi, Richard D. Schaller, Maryna I. Bodnarchuk, Dimos Poulikakos, Maksym V. Kovalenko

**Affiliations:** 1 Institute of Inorganic Chemistry, Department of Chemistry and Applied Bioscience, ETH Zürich, Zürich, Switzerland; 2 Laboratory for Thin Films and Photovoltaics, Empa – Swiss Federal Laboratories for Materials Science and Technology, Dübendorf, Switzerland; 3 Laboratory of Thermodynamics in Emerging Technologies, Zürich, Switzerland; 4 Swiss Center for Electronics and Microtechnology (CSEM), Landquart, Switzerland; 5 Department of Theoretical Physics Nanosystems, Chuiko Institute of Surface Chemistry of National Academy of Sciences of Ukraine, Kyiv, Ukraine; 6 Center for Nanoscale Materials, Argonne National Laboratory, Lemont, IL USA; 7 Department of Chemistry, Northwestern University, Evanston, IL USA

**Keywords:** Quantum optics, Nanoparticles

## Abstract

Traditional fluorescence-based tags, used for anticounterfeiting, rely on primitive pattern matching and visual identification; additional covert security features such as fluorescent lifetime or pattern masking are advantageous if fraud is to be deterred. Herein, we present an electrohydrodynamically printed unicolour multi-fluorescent-lifetime security tag system composed of lifetime-tunable lead-halide perovskite nanocrystals that can be deciphered with both existing time-correlated single-photon counting fluorescence-lifetime imaging microscopy and a novel time-of-flight prototype. We find that unicolour or matching emission wavelength materials can be prepared through cation-engineering with the partial substitution of formamidinium for ethylenediammonium to generate “hollow” formamidinium lead bromide perovskite nanocrystals; these materials can be successfully printed into fluorescence-lifetime-encoded-quick-read tags that are protected from conventional readers. Furthermore, we also demonstrate that a portable, cost-effective time-of-flight fluorescence-lifetime imaging prototype can also decipher these codes. A single comprehensive approach combining these innovations may be eventually deployed to protect both producers and consumers.

## Abstract (web summary)

Designing effective covert security features is highly regarded to deter counterfeit of goods and currency in the global markets. Here, the authors present an electrohydrodynamically printed unicolour multifluorescent-lifetime security tag system based on perovskite to provide an alternative yet affordable solution.

## Introduction

Counterfeiting or the production and sale of fraudulent goods is an increasingly prevalent global issue that affects companies, governments and consumers, with an estimated annual global cost of around EUR 530 billion^1,2^. The adverse effect of counterfeiting on the economy and society necessitates the constant development of anticounterfeiting approaches of ever-increasing complexity, typically employing novel materials and/or detection methods.

One ubiquitous example is fluorescent tags, which are widely used as security elements, and they are often encountered in banknotes, documents and luxury goods. Currently, fluorescent tags are simply checked by verifying their appearance (colour, pattern) under UV light by eye or with the aid of machine vision^3–9^. To further enhance security, additional covert features are required. One such technique that has recently attracted interest is the measurement of the fluorescence lifetime and, in particular, Fluorescence Lifetime Imaging (FLI). This approach relies on the ability to distinguish unicolour or spectrally overlapped features by their photoluminescent (PL) lifetimes and would add a crucial level of complexity related to material composition, synthetic control and code recognition.

Creating unicolour-lifetime-distinguishable features requires significant control over both photon emission energy and relaxation rate. While many inks based on fluorescent materials like quantum dots (i.e. strongly quantum-confined nanocrystals (NCs)) are lifetime-tunable^10,11^, the compositional or structural modifications that accompany these changes are often simultaneously coupled to changes in emission wavelength and quantum yield^12–14^. This observation is supported by Fermi’s golden rule, which generally indicates that the fluorescence lifetime is inversely proportional to the transition energy for a quantum oscillator^15,16^. This general limitation also applies to ABX_3_ (A = Cs, formamidinium (FA); B = Pb; X = Cl, Br, I) perovskites, a relatively new and emerging class of solution-processed semiconductors. Although the bandgap in these systems may be altered by halide composition and size^17–21^, achieving PL lifetime-distinguishable unicolour tags is nearly impossible with standard ABX_3_ compositions as a result of the same limitation by Fermi’s golden rule. Unlike traditional quantum dots of conventional semiconductors (e.g., CdSe, InP), lead-halide perovskites may offer alternative routes towards independent emission-lifetime balancing owing to their structurally soft and ionic lattices^22–24^, with much greater structural and compositional engineerability.

Here, we utilized structural engineering to further soften the lead-halide lattice by introducing large organic cations into perovskite nanocrystals (NCs) to circumvent the limitations imposed by Fermi’s golden rule. The use of bulkier organic cations such as ethylenediammonium {en}^25–29^, propylenediammonium and trimethylenediammonium^30^ for doping bulk and thin nanostructured films of ABX_3_ (where B is Pb or Sn) perovskites was recently demonstrated to controllably augment the bandgap, e.g. shifting the bandgap of FASnI_3_ from 1.43 eV to 1.48 V in {en}FASnI_3_ (10% substitution). In addition to the hypsochromic effect of {en} doping, these materials also exhibit increased PL-lifetimes^27,31^, demonstrating a behaviour that is opposite to that expected from Fermi’s golden rule. Given these promising observations, we sought to utilize this approach with the synthesis of {en}-doped perovskite NCs as an original route towards creating lifetime-differentiable unicolour inks.

For this approach, we took FAPbBr_3_ NCs as the starting material for developing a counter-ink to the CsPbBr_3_ NC ink. Since FAPbBr_3_ has the intrinsically smaller bulk-bandgap, the introduction of {en} will shift the bandgap towards higher energies to match that of CsPbBr_3_ while increasing the PL lifetime. The alternative approach of utilizing {en}CsPbBr_3_ NCs was not considered as this material could only match the bandgap of undoped CsPbBr_3_ NCs when the latter are produced as very small (strongly quantum-confined and hence suitably blue-shifted) and rather unstable NCs.

The implementation of these materials into fields such as the printing of security features requires both the preparation of colloidal NC inks and a patterning method able to quickly and precisely deposit several layers of materials at high-resolution with simple pattern reconfiguration. In general, additive manufacturing has provided a pathway towards inexpensive and flexible patterning while avoiding the additional development steps that accompany resist-based lithography^32^. Although inkjet printing has garnered attention in recent years due to its ability to print over large areas^33^, the practically attainable resolution of this method is limited to a few tens of micrometres with poor droplet-placement precision^34–36^. Alternatively, electrohydrodynamic (EHD) printing attains sub-micrometre resolution with droplets that are much smaller than the nozzle that they emanate from^37,38^. Herein, we combine the high-resolution capability of EHD nanoprinting with the specially engineered emissive properties of lead-halide perovskite NCs, to create millimetre-sized lifetime-encoded security tags and features.

The final hurdle to cost-effective lifetime-encoded security tags is finding a suitably inexpensive, simple, and portable detection method, alternative to time-correlated single-photon counting fluorescence lifetime imaging (TCSPC-FLI). One excellent contender to TCSPC-FLI is based on time-of-flight technology, which is currently used in several consumer electronic products where video capture with depth information is required^39,40^ and also has been considered as a technique for FLI recording^41–43^. This same ToF concept was recently applied to FLI imaging (ToF-FLI) to retrieve fast thermographic information where high temporal and spatial resolution were both necessary^44^.

This study presents three main findings: (i) cation-engineered perovskite NCs with nearly identical emission spectra, but vastly different PL lifetimes; (ii) EHD printed unicolour fluorescence-lifetime-encoded-quick-read (FLQR) codes; (iii) ToF-FLI decryption of FLQR codes. These three findings together represent the development of an advanced yet affordable anticounterfeiting system based on the fast and portable fluorescence lifetime imaging provided by ToF-FLI and the unicolour-multi-lifetime encoded fluorescence tags.

## Results

**Fig. 1:**
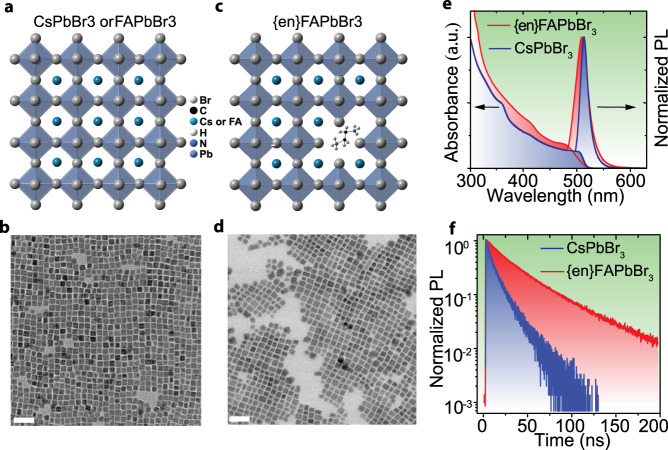
Colloidal perovskite NCs and their optical properties. Structural and morphological comparison of CsPbBr_3_ and {en}FAPbBr_3_. **a**, **c** Schematic of the structural difference between the undoped and {en}-doped (hence hollow) perovskite NCs; octahedral tiltings in CsPbBr_3_ are omitted for simplicity. **b**, **d** TEM images of CsPbBr_3_ NCs and {en}FAPbBr_3_ NCs (scale bar is 20 nm). **e** Absorption and PL spectra and **f** time-resolved photoluminescence traces for CsPbBr_3_ (blue) and {en}FAPbBr_3_ (red) NCs.

To maximize the spectral overlap and further enhance the difference between the PL lifetimes of our perovskite NC inks (CsPbBr_3_ and FAPbBr_3_), we utilized {en} doping in FAPbBr_3_ (Fig. 1). Colloidal {en}FAPbBr_3_ NCs were prepared through the addition of ethylenediamine diacetate during the synthesis (14% of {en} with respect to FA), while CsPbBr_3_ NCs were synthesized according to a previously reported procedure^17,45^. The obtained perovskite NCs are cuboid-shaped and relatively uniform for both CsPbBr_3_ (10 nm ± 1 nm; Fig. 1a, b) and {en}FAPbBr_3_ (11 nm ± 2 nm; Fig. 1c, d).

As a result of their similar band gaps (determined for perovskites by the metal-halide composition) and weak quantum confinement, both NC solutions of CsPbBr_3_ and {en}FAPbBr_3_ NCs exhibit excitonic absorption and narrow-band emission at nearly the same wavelength (Fig. 1e). The PL and absorption of {en}FAPbBr_3_ NCs are slightly blue-shifted (Supplementary Fig. 1a) compared to pure FAPbBr_3_ NCs^46^. Furthermore, the incorporation of the {en} results in the drastic increase in the PL lifetime (Supplementary Fig. 1b, Fig. 1f), namely from *ca*. 7 ns for CsPbBr_3_ NCs and 15 ns for FAPbBr_3_ NCs to 37 ns for {en}FAPbBr_3_ NCs.

The increase of the bandgap can be explained by the incorporation of the {en} cation into the perovskite structure, which results in both A-site cation substitution and the formation of Pb^2+^ and Br^-^ vacancies^25–27,31^. This leads to a reduction in the Pb-Br orbital overlap; the valence and conduction bands become flatter and the bandgap increases. Characterization of these {en}FAPbBr_3_ NCs by x-ray powder diffraction (XRD, Supplementary Fig. 1c and 1d), shows an expected expansion of the unit cell parameters compared to the undoped materials and confirms incorporation of {en}. We attribute the drastic increase in the measured PL lifetime of {en}FAPbBr_3_ NCs to the alteration of the intrinsic energy levels and structural dynamics (exciton-phonon coupling) that are both induced by the incorporation of {en}. An alternative explanation could be the decrease in bulk and surface trap densities and hence decreased contribution of faster non-radiative channels.^25,27,31^ This latter scenario can be ruled out since PLQYs are comparable for {en}FAPbBr_3_, CsPbBr_3_ and FAPbBr_3_ NCs in solution (75–90%), where {en}-doping slightly decreases PLQY to a minimum of 75–80%.

We then set about to comprehensively characterize these novel “hollow” {en}FAPbBr_3_ perovskite NCs with both temperature- and time-resolved emission spectroscopy as well as transient absorption (TA) and compare the results with those of undoped FAPbBr_3_ and CsPbBr_3_ NC counterparts. The steady-state absorption spectra (Fig. 1e and Supplementary Fig. 1a) already suggest that {en}-doping extends the interval for the excitonic transition, which can be seen as a broadening of the absorption edge. The derivatives of the steady-state absorption band widths (Supplementary Fig. 2a) are corroborated by the bleach bands in the transient absorption spectra (Supplementary Fig. 2b). The retention of a high degree of monodispersity among NCs (Fig. 1b, d) as well as their large size (weak confinement regime) exclude inhomogeneous broadening as a contributing factor. We therefore interpret the smeared absorption features as a result of homogeneous broadening due to local structural deformations created by {en} doping, which may result in multiple allowed transitions. This effect alone leads to an increase in PL lifetime, which is supported by a quantum-mechanical calculation relating the number of electronic transitions to the radiative lifetime (using the effective mass approximation; Supplementary Note 1).

**Fig. 2:**
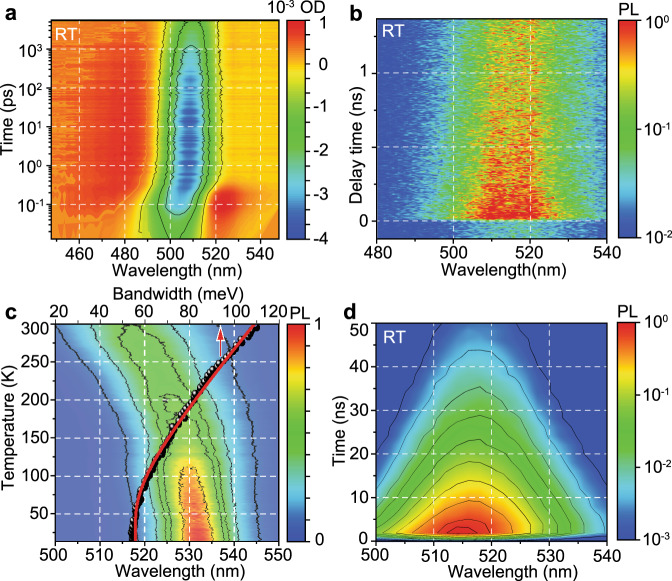
Optical spectroscopy characterization of {en}FAPbBr3 NC film. 2D pseudo-colour plots for fast decay range: **a** transient absorption, 0–5 ns and **b** transient PL, 0–1.5 ns of; **c** 2D pseudo-colour plot of temperature dependence of PL emission, data points and fitting curve (abscise axis is on top) – bandwidth parameter of Lorentzian fit for the PL spectra at the corresponding temperature; **d** 2D pseudo-colour plots for time-resolved PL for long decay range, 0–50 ns.

Apart from the different spectral widths of both the TA bleach and PL emission bands, transient absorption spectra of {en}FAPbBr_3_, FAPbBr_3_ and CsPbBr_3_ NCs are rather similar, all showing fast initial carrier cooling (0–1 ps), photoinduced bleach bands for delay times in the range of 1 ps–2 ns (Fig. 2a and Supplementary Fig. 3), and a slight time-delayed redshift in PL emission (data recorded with Streak-camera, Fig. 2b and Supplementary Fig. 4).

Upon further investigation, we observed that by cooling the {en}FAPbBr_3_ NC film a monotonic temperature-dependent redshift of the PL band occurs (Fig. 2c; a general trend for 3D metal halide perovskites). In addition, the cooling results in the narrowing of the PL emission band with the bandwidth (Lorentzian-fitted from the PL line shape, data points and a line on Fig. 2c) decreasing from 110 meV to 55 meV upon cooling to 12 K. This temperature variation of the bandwidth suggests a significant role for exciton–phonon interactions and reduced lattice rigidity. Namely, the “hollow” structure results in a softer lattice that promotes large polaron screening, which is known to occur in metal halide perovskites^22–24,47^. The impact of polarons might be phenomenologically described by an increase in effective carrier masses^48^; this leads to a decrease in the exciton binding energy and thus a lengthening of the PL lifetime^49^. At the same time, the large residual PL broadening (about 55 meV) at 12 K can be attributed to either the presence of multiple allowed excitonic transitions, as mentioned earlier, or to the inhomogeneous doping of {en} in {en}FAPbBr_3_ NCs. The hypothesis of multiple radiative transitions is supported by a comparison of the time-resolved PL traces (TCSPC-acquired) for 3 different perovskite NC inks (Supplementary Fig. 5). Rather than compare the spectral maximum of emission for these traces, we chose to compare their lower and higher energy wings (based on 50% of max PL intensity, i.e. full-width-at-half-maximum (FWHM) range boundaries, and as sketched in the insets of Supplementary Fig. 5) in order to highlight the multilevel interaction. A peculiar build-up feature was observed within 0–3 ns in the longer wavelength PL emission trace (red), specifically, for the {en}FAPbBr_3_ NC film. This delay suggests a cascade-like relaxation process in which several excited state levels are populated and contribute to the overall emission process. While the blue and red wavelength portions differ in the presence of a build-up time after excitation, these traces exhibit similar decay behaviour afterwards (Supplementary Fig. 5c). When such time-resolved PL spectra are viewed in a 2D pseudo-colour plot (time vs. wavelength), this behaviour appears spectrally symmetric with respect to the PL max (Fig. 2d). This is corroborated by previous data on the spectrally symmetric decay from intrinsic excitonic emission for a perovskite single crystal in ref. ^50^, where surface defects would instead demonstrate a significant time-delayed redshift in PL emission.

Cooling {en}FAPbBr_3_ NCs to 12 K reveals a drastic increase in the variation in PL lifetime with emission wavelengths in the FWHM range (Supplementary Figs. 6 and 7). More specifically, RT traces exhibit very little variation (from 27 to 31 ns, or 15%) whereas traces at 12 K vary from 1 to 3 ns (300%). At room temperature, the phonon landscape assists in the repopulation of upper levels thus effectively averaging their PL emission lifetimes. At lower temperatures with the freezing out of the majority of phonons, the multiple levels generated by local structural deformations become individually expressed in the PL lifetime spectrum. This is related to the presence of a strong phonon-mediated multi-level interaction, which is enhanced by the softer lattice of the {en}FAPbBr_3_ NCs. Therefore cation-engineering, through the use {en} doping, allowed us to create materials with strongly varying PL lifetimes and unicolour emission, satisfying a critical material requirement for such FLQR security tags.

**Fig. 3:**
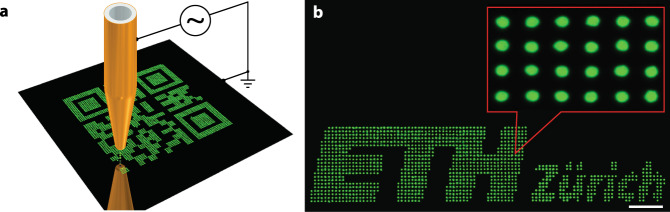
EHD printing. **a** Scheme representing the EHD printing setup used to print the colloidal perovskite CsPbBr_3_ NC inks. A voltage is applied between a gold-coated capillary nozzle and a grounded substrate, which drives the EHD ejection of ink droplets. **b** An example of the high-resolution printed pattern (~12,000 dpi) showing low dot-to-dot variation, the distance between dots is 2 µm, the scale bar is 20 µm.

Although possessing high-quality unicolour inks is a necessary part of producing fluorescent lifetime security tags, the utilization of this unique characteristic for security applications requires a deposition technique that offers high spatial resolution, compatibility with arbitrary substrate topologies, scalability and the absence of chemical or solvent-based treatments that are known to degrade and destroy perovskite-based materials^51,52^. In order to showcase the perovskite-based-lifetime-encoded approach, a two-layer method of concealing the code was necessary. The first layer would contain the QR code, and the second layer would consist of a masking ink placed in the empty spaces of the QR code. This feature imposed strict requirements on the printing resolution and alignment that are not achievable with standard ink-jet printing technique. Consequently, after examining several printing methods, EHD printing was determined to be the method of choice (see Supplementary Note 2). In EHD printing, a pulsed electric field is used to eject attoliter sized droplets from the nozzle tip and direct them onto the substrate with high droplet placement accuracy (Fig. 3a, Supplementary Fig. 8a, b and Supplementary Note 3)^37,53–55^. These features were validated using a single ink (CsPbBr_3_ NCs in 1,3,5-triethylbenzene), which could be printed at a resolution of over 10,000 dpi while maintaining high dot-to-dot uniformity and the bright PL of the initial solution (Fig. 3b; Supplementary Movie 1).

**Fig. 4:**
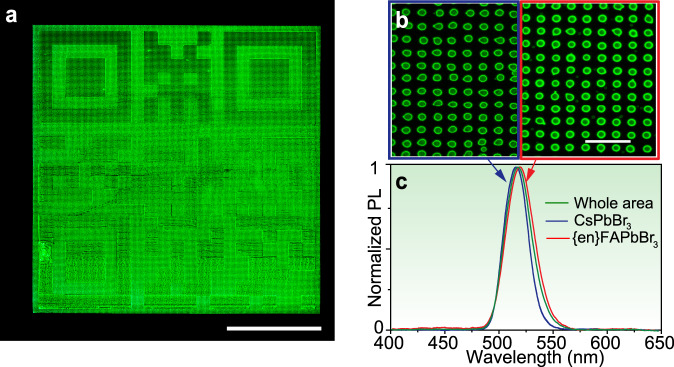
Unicolour FLQR tags. **a** A true-colour image of unicolour FLQR code that was printed as two separate images (QR code and its inverse) from perovskite NC inks (scale bar is 800 µm); **b** zoomed-in image of **a** containing regions of CsPbBr_3_ (blue) and {en}FAPbBr_3_ (red) inks (scale bar is 30 µm). **c** The spatially resolved PL spectra from the corresponding regions of (blue and red rectangles on b), whose PL spectra overlap to form one apparent emission band (green).

FLQR codes require the accurate deposition of two separate inks to be printed successively as a QR code and its inverse in order to hinder the unauthorized machine-reading of the encoded information with ordinary PL-emission based cameras. To realize this concept, a QR code was printed using one of the NC inks (CsPbBr_3_ or {en}FAPbBr_3_), while its complementary, inverse image was printed with the other (Fig. 4a). Both inks were prepared by dissolving the quantum dots in 1,3,5-triethylbenzene (~25 mg/mL; Supplementary Note 4). To print, two different capillary nozzle print heads were aligned with respect to one another and used sequentially – one for each ink (the alignment procedure of the two inks is detailed in the Supplementary Fig. 8c and Supplementary Note 5). The resulting high degree of alignment (Fig. 4b) and spectral overlap (Fig. 4c) almost completely exclude the identification of the individual sub-patterns (marked blue for CsPbBr_3_ and red for {en}FAPbBr_3_). Despite local variations in emission intensity, and the relatively small shift between arrays, the FLQR code cannot be easily deciphered and is not machine-readable under steady-state excitation using ordinary RGB cameras.

**Fig. 5:**
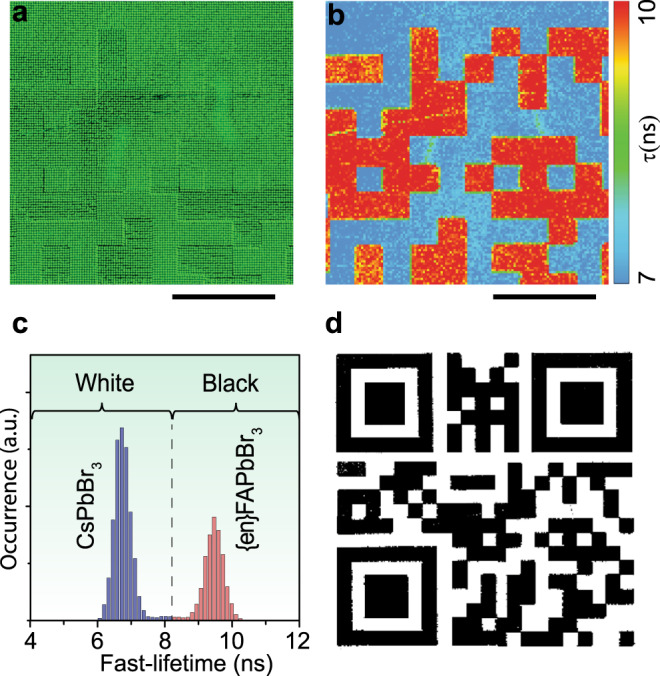
FLI microscopy (FLIM) imaging and binarization of FLQR tags. **a** Zoomed-in view of the central portion of the unicolour FLQR code printed with CsPbBr_3_ and {en}FAPbBr_3_ inks. **b** TCSPC-FLI microscopy pseudo-colour image of the same region (scale bars are 400 µm). **c** The two inks exhibit two distinct fast-lifetime (see Supplementary Note 6) histograms that can be defined as white (lifetime ~6.5 ns, blue) and black (lifetime ~9.5 ns, red) to create a machine-readable QR code **d** (pattern size is identical to that in Fig. 4a). Please try to scan it with your smartphone.

These previously unreadable FLQR codes can indeed be read through the use of fluorescent lifetime imaging techniques. This, however, requires the use of a rather expensive (>$300k) confocal microscope system coupled to TCSPC-FLI or high-speed time-gated camera unit with a pulsed picosecond laser as the excitation source. Due to the limited field-of-view of the confocal microscope, we first analyzed the central tile of the pattern where the PL intensities of the two inks were roughly equivalent (Fig. 5a). By examining the FLI image, the two distinct lifetimes of the CsPbBr_3_ and the {en}FAPbBr_3_ inks were easily identified and pseudocolour mapped to reveal the otherwise hidden pattern (Fig. 5b). These lifetimes are shorter than those measured in solution, and this is likely the result of the printing process as the most-suitable solvent for printing (Supplementary Note 4) is not an ideal solvent for perovskite nanocrystals. This discrepancy can be measured when comparing films drop-casted from toluene and those prepared by EHD printing (Supplementary Fig. 9a). However, these effects do not prevent the ability to measure and sufficiently binarize the results to decipher the printed, lifetime-encoded tags, regardless of the procedure used to estimate the lifetime: fast-lifetime (1/e decay parameter) vs. average lifetime from a biexponential fit (see Supplementary Fig. 9b; Supplementary Note 6). This is not yet, however, a machine-readable code. Rendering it as an identifiable code required an intermediate binary discretization step in which the bimodal lifetime distribution was binned as either “white” (fast lifetime, CsPbBr3) or “black” (slow lifetime, {en}FAPbBr3) using a threshold value of 8.2 ns (Fig. 5c). Redrawing the whole FLQR code after this binary discretization yields a machine-readable FLQR code that can be read by any smartphone with QR-reading software (Fig. 5d; the QR code provides a link to this article).

**Fig. 6:**
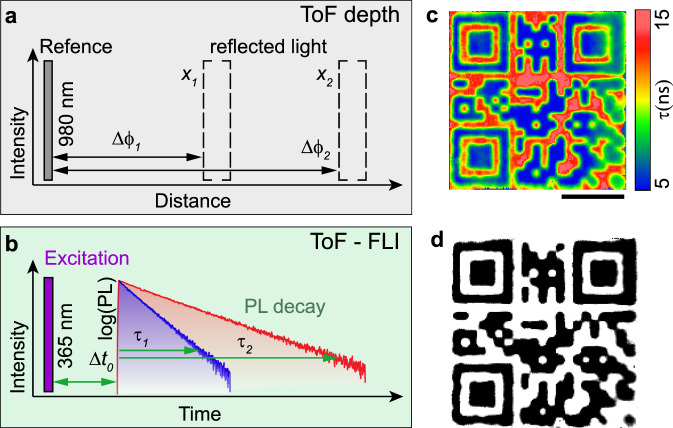
ToF-FLI principle and imaging. **a** Principle for ToF-based LIDAR where *x* is distance and *Δϕ* is the corresponding change in phase. **b** The analogous approach for ToF-based TRPL where *Δϕ* is replaced by the fluorescent lifetime, *τ*. **c** Lifetime pseudo-colour map of FLQR as recorded by the ToF-FLI imager (scale bar is 1 mm). **d** Black-and-white machine-readable QR code after binary-discretization of (e), Please try to scan it with your smartphone.

Recognizing economic aspects as crucial factors that limit the adoption of this new approach towards securing the trade of consumer goods, we sought a less expensive yet faster alternative to TSCPC-FLI microscopy. The recent and rapid development of ToF-based LIDAR technologies (Fig. 6a) such as those found in self-driving cars and Kinect^TM^ products inspired us to develop a prototype lifetime imager based on this same ToF-principle (Fig. 6b–d), which we have previously demonstrated for recording thermographic images with low-dimensional perovskite luminophores (exhibiting PL lifetimes of hundreds of nanoseconds to several microseconds)^44^. Briefly (detailed description is in Supplementary Notes 2–4 of ref. ^44^), this concept, which is used in range determination (*x*), is based on the measurement of phase differences (*Δϕ*) due to a delay in the propagation of reflected light with respect to a reference pulse (ToF-depth; Fig. 6a). This principle was recently applied to PL lifetime (*τ*) determination using the delay between excitation and emission light (ToF-FLI; Fig. 6b). The read-out and successful recognition process with the ToF-FLI imager requires pre-calibration of zero phase shift with a non-emissive, light scattering sample and an absent excitation-blocking long-pass optical filter. The excitation modulation frequency should be adopted to the particular materials used in the FLQR code in order to obtain maximal contrast in the phase-shifted image. For the {en}FAPbBr_3_ and CsPbBr_3_ NCs, the best contrast was achieved with 4 MHz modulation. Also, both intensity-saturated and very dim areas should be avoided during analysis. Consequently, FLQR codes must be printed with uniform intensity, and codes must be measured with optimized exposure settings to ensure best results. Utilizing the cost-effective approach of a harmonically modulated excitation source, these differences are calculated from 4 points (denoted *I*_*1*,_ *I*_*2*_*, I*_*3*,_ *I*_*4*_) at 4 phase-locked positions (0°, 90°, 180°, 270°) which are then recalculated into a corresponding lifetime using the modulation depth (*M*), the average intensity (*I*), and the phase delay (*Δϕ*; Supplementary Fig. 10a). Not only does this method exclude the use of expensive, picosecond pulsed laser sources and optoelectronics capable of single-photon counting, but it also allows for fast, one-shot image recording using a pulsed LED and a CMOS ToF imaging array (Supplementary Fig. 10b). For example, acquisition times for TCSPC-FLIM were about 15–30 min whereas the exposure time for the ToF camera was about 2 s. In addition to this, the device is compact and lightweight making it a promising candidate for future commercialization (Supplementary Fig. 10c, d).

To validate this approach and demonstrate its robustness, we imaged a similar FLQR code that was adopted to a larger field-of-view (Fig. 6c). This FLQR code was printed in an inverse manner (QR code pattern with CsPbBr_3_ and the complimentary pattern using {en}FAPbBr_3_) to demonstrate that ToF-based binary discretization could also be applied to these FLQR tags to yield the final, smartphone-readable QR code (Fig. 6d). Although this final QR code is relatively worse in quality and resolution than the one obtained by TCSPC imaging (Fig. 5d), it demonstrates that such an inexpensive and lightweight prototype is indeed able to compete with established state-of-the-art methods. As ToF-based technologies advance in the consumer market, we expect our ToF-FLI method to improve in maximum spatial resolution, capture rate, and dynamic range while becoming lower-cost and more portable.

In summary, we have presented an innovative and comprehensive anti-counterfeiting solution through the cation-engineering of perovskite inks with the synthesis and characterization of “hollow” {en}FAPbBr_3_ NCs, the utilization of high-resolution printing, and the implementation of a ToF-FLI device for deciphering FLQR tags. The combination of hollow and undoped perovskite-based inks represents a reconfigurable system that offers nearly independent emission colour and lifetime tunability, through the variation of anions, cations, and the degree of quantum confinement. With these inks, we could demonstrate that final, machine-readable QR outputs could be created using EHD printing with scalability potentially down to 5 × 10^−4^ mm^2^. At the same time, laser-free ToF-FLI offers an affordable yet portable method for decoding and validating lifetime-encoded tags.

## Methods

### Materials

Chemicals: lead (II) acetate trihydrate (>99.99%), hydrobromic acid (HBr, 48%), formamidine acetate (99%), ethylenediamine diacetate (98%), 1-octadecene (ODE, 90%), oleic acid (OAc, 90%), methyl acetate (>99.8%), and toluene (99.8%), Didodecyldimethylammoniumbromid (DDAB, 98%) were purchased from Sigma-Aldrich. Oleylamine (OAm, 95%) was purchased from STREM Chemicals. 1,3,5-triethylbenzene (>90%) was purchased from TCI. Ethanol (absolute, >99.8%) was purchased from VWR. Diethyl ether (>99.8%) was purchased from Fisher Scientific.

All chemicals were used as purchased without further purification unless otherwise noted within the procedure.

### Synthesis of CsPbBr_3_ NC ink

First, a Cs-oleate precursor solution was prepared by mixing Cs_2_CO_3_ (815 mg, 2.50 mmol) with ODE (40 mL) and OAc (2.5 mL) in a 100 mL flask. The mixture was degassed three times at RT, heated to 100 °C for 30–60 min, and then left to cool to RT. The solution was stored in a glovebox. For the synthesis of CsPbBr_3_ NCs, lead (II) bromide (1.10 g, 3.00 mmol) was mixed with ODE (100 mL) in a 500 mL flask and stirred at 1000 rpm. The mixture was heated to 100 °C, dried for 20–30 min and then heated to 180 °C under a nitrogen atmosphere. When a temperature of ca. 120 °C was reached, OAc (20 mL) and OAm (20 mL) were injected. At 180 °C, 16 mL of the caesium oleate solution was injected from a 100 mL-dropping funnel under vacuum (<10 mbar). After 15 s the reaction mixture was cooled with an ice bath. The crude solution was centrifuged for 5 min at 12.1k rpm. The resulting supernatant was discarded and the precipitate was dispersed in hexane (6 mL). The solution was centrifuged a second time for 1 min at 10k rpm and the precipitate was discarded. The supernatant was isolated and diluted with additional hexane (6 mL). DDAB-treatments of NCs was conducted by mixing 6 mL of NCs in hexane, 6 mL toluene and 1.56 mL of the DDAB/toluene solution (0.05 M) and stirring it for 1 h. The mixture was then washed by adding 18 mL ethyl acetate, followed by centrifugation at 12.1k rpm for 5 min. The supernatant was discarded and the precipitate was dispersed in 6 mL toluene. CsPbBr_3_ NCs were solubilized in 1,3,5-triethylbenzene for printing. In a typical solvent exchange procedure, 1 mL of 1,3,5-triethylbenzene was added to 1 mL of the NC toluene solution. The toluene was then evaporated under a moderate vacuum. Concentrations of the CsPbBr_3_ solution used for printing were around 25–28 mg/ml.

### Preparation of {en}FAPbBr_3_ NC ink

First, oleylammonium bromide was prepared by reacting cold solution of OAm (12 ml) in ethanol (100 ml) with hydrobromic acid (9 ml, 48%, added dropwise). The solution was dried in a rotary evaporator under vacuum at 50 °C, washed multiple times with cold diethyl ether, and the white powder was then isolated by vacuum-drying at 70 °C. {en}FAPbBr_3_ NCs were synthesized by adapting the previously published synthesis of FAPbBr_3_ NCs^46^. In a 25 ml 3-neck flask, Pb(acetate)_2_ ∙ 3H_2_O (76 mg, 0.2 mmol), formamidine acetate (78 mg, 0.75 mmol) and ethylenediamine diacetate (18.1 mg, 0.1 mmol) were combined with ODE (8 ml, pre-vacuum-dried at 100 °C) and OAc (2 ml, pre-vacuum-dried at 100 °C). After drying under vacuum at 50 °C for 40 min, the mixture was heated under nitrogen to 130 °C. At this point, the solution of oleylammonium bromide (266 mg, 0.75 mmol) in toluene (2 ml) was swiftly injected. After 30 s, the reaction mixture was cooled in an ice-water bath. The NCs were purified by adding methyl acetate (7 ml) to the crude solution, followed by centrifugation at 12.1k rpm for 3 min. The precipitate was dispersed in 5 ml toluene, the dispersion was again centrifuged at 5k rpm for 3 min, and the final precipitate was discarded. For printing, the solvent was changed by combing 1.95 ml of supernatant, OAc (0.06 ml) and 1,3,5-triethylbenzene (1.65 ml), and then removing the toluene by evaporation under vacuum.

### EHD high-resolution ink-jet printing

Printing nozzles were fabricated by pulling borosilicate capillaries (World Precision Instruments TW100‐4) to an outer diameter of ~2–3 µm (Sutter Model P‐97). The capillaries were then rendered conductive by a thin coating of Ti/Au (5 and 50 nm) via e-beam evaporation. To print, the nozzles are back-filled with an ink and moved to a working distance ~10 μm from the substrate. The sample is positioned on a conductive grounded plate and electrohydrodynamic ejection is induced by applying a square wave voltage of 250–350 V_p_ at 1 kHz to the nozzle. All printing was performed under ambient conditions (23 °C, 30–45% relative humidity). The printing setup used for this work is equipped with a confocal laser microscope for in-situ visual inspection. The pulse length, voltage applied to the nozzle, and stage position are controlled using a custom-built control unit.

### TCSPC-FLI and confocal microscopy

TCSPC-FLI measurements and confocal microscopy was carried out on a Leica TCS SP8 confocal microscope equipped with a pulsed diode 440 nm laser (PDL 800-B), 10x objective (0.4NA), and a PicoHarp 300 TCSPC Module.

### ToF-FLI

The ToF-FLI prototype includes a modulated light source (LED, 365 nm emission wavelength), a CMOS ToF imager (256 × 256 pixels) originally developed by CSEM for 3D imaging, dedicated FPGA-based electronics, and optical components for illuminating the probe and collecting the PL emission. The camera electronics are based on a stacked PCB approach, including a base board, a FPGA processing module and a sensor head PCB. An external PC (connected via USB protocol) is used to set the measurement parameters (such as modulation frequency, illumination intensity and integration time) and display the results. The LED modulation frequency can be varied between 3 kHz and 20 MHz, allowing the measurement of PL lifetimes from hundreds of microseconds down to a few nanoseconds with sub-nanosecond precision. With the current optics an area of 5.3 × 5.3 mm is imaged. The emission wavelength can be selected by exchangeable spectral filters. More detailed technical characteristics of the ToF-FLI image sensor setup are listed in the References. ^44,56^.

## Supplementary information

Supplementary Information

Description of Additional Supplementary Files

Supplementary Movie 1

## Data Availability

The data that support the findings of this study are available from the corresponding authors upon reasonable request.
