# Supplementary material for: Radiative lifetime-encoded unicolour security tags using perovskite nanocrystals
Source: Nat Commun. 2021 Feb 12;12:981. doi: 10.1038/s41467-021-21214-3 (PMC7881120; doi:10.1038/s41467-021-21214-3)
Supplement: Supplementary file 1 — Supplementary Information [file 41467_2021_21214_MOESM1_ESM.pdf]

Supplementary Information

Radiative lifetime-encoded unicolour security tags using perovskite nanocrystals

Sergii Yakunin,[1,2#] Jana Chaaban,[3#] Bogdan M. Benin,[1,2] Ihor Cherniukh,[1,2] Caterina Bernasconi,[1,2] Annelies Landuyt,[1,2] Yevhen Shynkarenko,[1,2] Sami Bolat,[2] Christoph Hofer,[4] Yaroslav E. Romanyuk,[2] Stefano Cattaneo,[4] Sergey I. Pokutnyi,[5] Richard D. Schaller,[6,7] Maryna I. Bodnarchuk,[2*] Dimos Poulikakos[3*] and Maksym V. Kovalenko[1,2*]*

[1] Institute of Inorganic Chemistry, Department of Chemistry and Applied Bioscience, ETH Zürich, CH-8093 Zürich, Switzerland

[2] Laboratory for Thin Films and Photovoltaics, Empa – Swiss Federal Laboratories for Materials Science and Technology, CH-8600 Dübendorf, Switzerland

[3] Laboratory of Thermodynamics in Emerging Technologies, ETH Zürich, Sonneggstrasse 3, 8092 Zürich, Switzerland.

[4] Swiss Center for Electronics and Microtechnology (CSEM), Center Landquart, CH-7302 Landquart, Switzerland

[5] Department of Theoretical Physics Nanosystems, Chuiko Institute of Surface Chemistry of National Academy of Sciences of Ukraine, 17 General Naumov Str., UA, Kyiv, 03164, Ukraine

[6]Center for Nanoscale Materials, Argonne National Laboratory, Lemont, IL 60439, USA.

[7]Department of Chemistry, Northwestern University, Evanston, IL 60208, USA

# Equally contributing authors *Corresponding authors

[*] E-mails: yakunins@ethz.ch, maryna.bodnarchuk@empa.ch, dpoulikakos@ethz.ch, mvkovalenko@ethz.ch

Table of Contents

Supplementary Figure 1. Optical and XRD characterization of FAPbBr3 and {en}FAPbBr3 NCs. 3

Supplementary Figure 2. Broadening of the excitonic transition 4

Supplementary Note 1. Evaluation of the effect of band-edge broadening on the exciton relaxation lifetime. 4

Supplementary Figure 3. 2D pseudo-colour plots for RT transient absorption of NCs colloidal solutions 8

Supplementary Figure 4. 2D pseudo-colour plots for Streak-camera –recorded RT transient PL for NCs films 8

Supplementary Figure 5. Comparison of TCSPC-acquired time-resolved PL traces for NCs films 8

Supplementary Figure 6. 2D pseudo-colour plots for temperature variation (12 K-300 K) of TCSPC-aquired time-resolved emission spectra 9

Supplementary Figure 7. Temperature variation (12 K-300 K) of a dependence (within central FWHM spectral range) for PL lifetime 10

Supplementary Note 2. Advantages of EHD printing over traditional ink-jet printing. 10

Supplementary Note 3. EHD printing procedure. 11

Supplementary Note 4. Selection of 1,3,5-triethylbenzene as optimal solvent for EHD printing: 12

Supplementary Figure 8. Printing alignment. Fluorescence microscope image 13

Supplementary Note 5. Alignment procedure for EHD printing with two inks. 13

Supplementary Figure 9. Difference in lifetime estimation. 14

Supplementary Note 6. Difference between the evaluation of 1/e decay time (fast-lifetime) and averaged lifetime. 14

Supplementary Figure 10. ToF-FLI prototype 15

Supplementary References 16

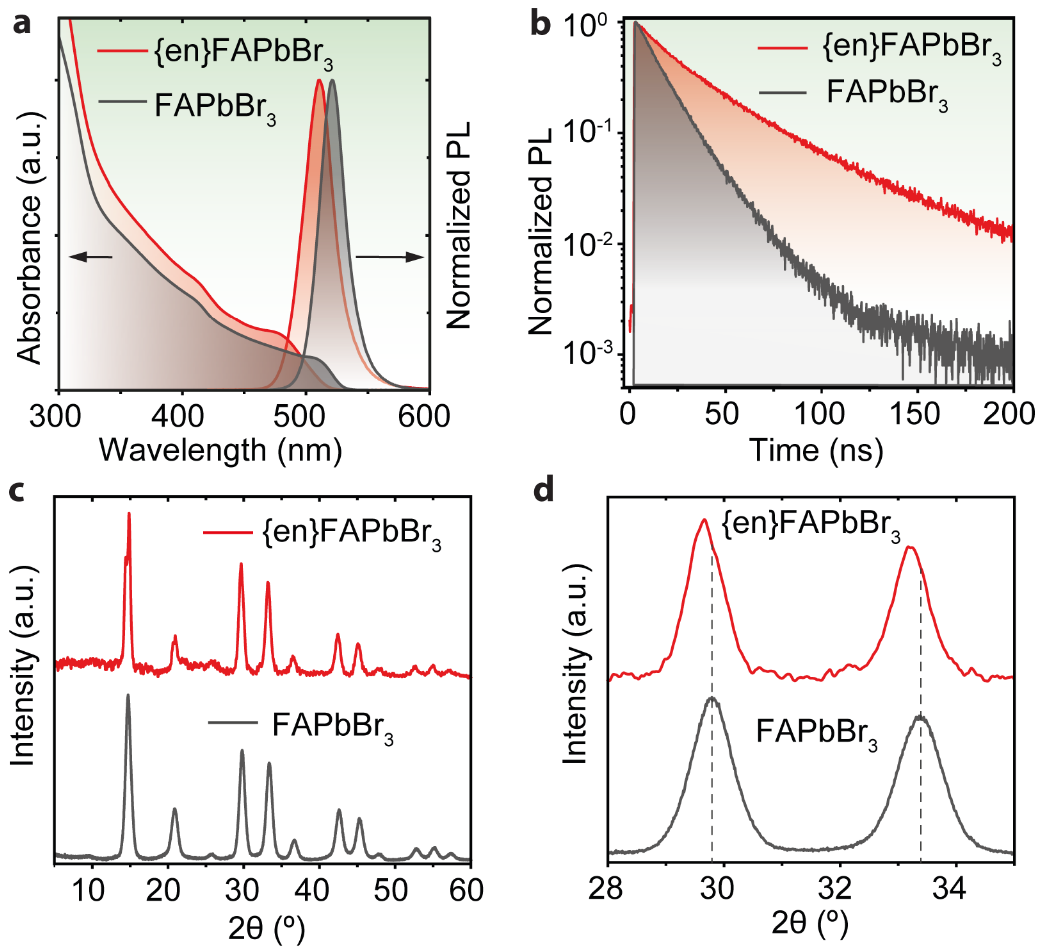

Supplementary Figure 1. Optical and XRD characterization of FAPbBr3 and {en}FAPbBr3 NCs. (a) Absorption and PL spectra for FAPbBr3 (gray) and {en}FAPbBr3 (red) NCs; (b) Time-resolved photoluminescence traces; (c,d) XRD patterns of FAPbBr3 and {en}FAPbBr3 NCs.

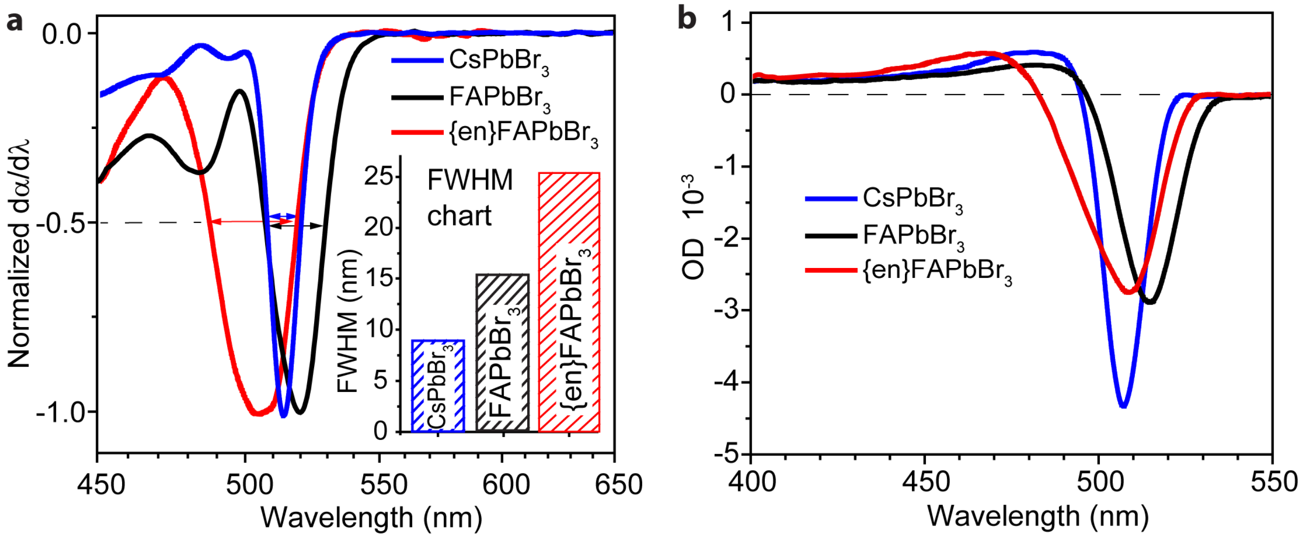

Supplementary Figure 2. Broadening of the excitonic transition for {en}FAPbBr3 (red) in comparison for CsPbBr3 (blue) and FAPbBr3 (black); (a) spectrum of the first derivative of the absorption spectra $\frac{d\alpha}{d\lambda}$ reveals the interval of the band-egde; (b) transient absorption spectra (see Supplementary Figure 3) integrated for the first 5 ns after the excitation pulse.

Supplementary Note 1. Evaluation of the effect of band-edge broadening on the exciton relaxation lifetime.

From experiments we know bandgaps values for NCs as well as the absorption edge broadening for all 3 types of NCs studied: ΔE_{edge}= 37 meV for $CsPbBr_3$, 71 meV for $FAPbBr_3$, and 120 meV for {en}$FAPbBr_3$. Furthermore, we observe that all types of these perovskite NCs have a size of about 11 nm and can be roughly approximated as being spheres with a 5.5 nm radius. The basic parameters are described in the Supplementary Table 1 below:

Supplementary Table 1. Physical parameters for perovskite NCs.

Perovskite NC composition	m_e, (m_0)	a_e, (nm)	m_h, (m_0)	a_h, (nm)	μ_{ex}, (m_0)	a_{ex}, (nm)	ε_{opt}	E_g *bulk*, (eV)	E_g NCs, (eV)	Size, (nm)
$CsPbBr_3$	0.15[1]	1.75	0.14[1]	1.88	0.072-0.126[1,2]	3.65	7.3[3]	2.34-2.38[3]	2.43	10 ± 1
$FAPbBr_3$	0.26	1.75	0.26	1.75	0.13[4,5]	3.5	8.6[3,4]	2.27-2.31[3,4]	2.34	11 ± 2
{en}$FAPbBr_3$	0.21	1.75	0.21	1.75	0.105	3.5	7*	2.341	2.43	11 ± 2

*The introduction of {en} cations creates structural porosity, and the effect of this structural porosity in {en}$FAPbBr_3$ results in the decrease of the effective dielectric constant down to 7.1, according to the Maxwell Garnett approximation, with an expected porosity of 15%.

Where ε is a dielectric constant; m_e and m_h are electron and hole masses, respectively; exciton reduced mass is calculated as $\mu_{ex} = m_e\, m_h/(m_e + m_h)$; the Bohr radii are $a_e = (\varepsilon\, \hbar^2/m_e\, e^2)$, for an electron; $a_h = (\varepsilon\, \hbar^2/m_h\, e^2)$, for a hole; and $a_{ex} = (\varepsilon\hbar^2/\,\mu_{ex}\, e^2)$, for an exciton. Since the average radii of the NCs are considerably larger than the Bohr radii of the electron and hole:

$$a_{h,}\, a_e < a \tag{1}.$$

then, we can assume that Eq (1) is valid and that the Coulomb interaction $V_{eh}(r) \approx (e^2/\varepsilon a)$ might be neglected[6]. This allows us to describe the electron and the hole in the NC with quantum numbers (n_e, l_e) and (n_h, l_h), respectively, using a spherical potential well with infinitely deep walls as a model[6,7]. Such confinement in the NCs leads to the origin of the allowed energy levels. Their energies are defined as:

$$E_{n,l}^{e(h)}(a) = \frac{\hbar^2}{2m_{e(h)}\, a^2}\, (\varphi_{n,l})^2, \tag{2}$$

where the subscripts (n, l) are the principal and azimuthal quantum numbers for the electron (hole); $\varphi_{n,l}$ are the roots of the Bessel function, *i.e.* $J_{l+1/2}(\varphi_{n,l}) = 0$. The energy levels $E_{n,l}^{e}(a)$ and $E_{n,l}^{h}(a)$ for an electron (n_e, l_e) and a hole (n_h, l_h), respectively, are located within the conduction band and valence band, correspondingly and described by equation (2). The effective broadening of the absorption edge in $FAPbBr_3$ NCs is formed by the electron transition between two quantum levels:

from (n_e=1,l_e=0) to (n_e=1,l_e=1), and from (n_e=1,l_e=1) to (n_e=1,l_e=2) with transition energies $\Delta E_{1,0}^{1,1}(a) = 51.2$ meV and $\Delta E_{1,1}^{1,2}(a) = 65.4$ meV. The doping affects the structure deformation and lattice softening in {en}$FAPbBr_3$ NCs and results in three allowable transitions: (n_e=1,l_e=0) to (n_e=1,l_e=1), (n_e=1,l_e=1) to (n_e=1,l_e=2) and (n_e=1,l_e=2) to (n_e=1,l_e=3) with transition energies $\Delta E_{1,0}^{1,1}(a) = 64$ meV, $\Delta E_{1,1}^{1,2}(a) = 82$ meV, and $\Delta E_{1,2}^{1,3}(a) = 112$ meV correspondingly.

In the frequency range $\omega_{n,l}\,(a) = \ E_{n,l}(a)/\hbar$, which corresponds to the above-considered states of charge carriers in a NC, (n, l) (2), the wavelength of light is much larger than the dimensions of these states $(\sim a_e, a_h\ )^4$. Therefore, the behavior of these states is adequately described by the dipole approximation. In this case, the operator of the dipole moment of the electron (hole) located in the NC bulk is expressed as[8]:

$$\mathbf{D}(\mathbf{r}) = \beta\, e\, \mathbf{r}, \quad (3)$$

where **r** is the radius vector that determines the distance between the electron and the center of the NC, $\beta\ =\ 3\varepsilon_0/2(\varepsilon_0 + \varepsilon)$, and ε_0 is the permittivity matrix ($\varepsilon_0 =\ 1$).

To estimate the value of the dipole moment, it is sufficient to consider the transition between the lowest quantum-confined states (2), *e.g.*, between the states (n_e,l_e) and (n_e,l_e+1) for an electron. The states are described by wave functions in an infinitely deep spherical potential well. To calculate the matrix element of the dipole moment of the charge-carrier transition from the (n_e, l_e) state to the (n_e,l_e+1) state, $D_{n,l}^{n,l+1}(a)$, we assume that the uniform field of the light wave $\varepsilon(\omega,t)$ is directed only along the Z-axis (ω is the wave frequency). In this case, we take the dipole moment, **D**(**r**) (4), induced by the field, $\varepsilon(\omega,t)$, as the perturbation responsible for such a dipole transition. The expression for the dipole moment of the transition, $D_{n,l}^{n,l}(a)$, follows from formula (3):

$$D_{n,l}^{n,l}(a) =< n_e, l_e|\ \mathbf{D}(\mathbf{r})|n_h, l_h > \quad (4)$$

If condition (1) is satisfied, we can then use, for the electron states (n_e,l_e) and (n_e,l_e+1) the wave functions in a NC as a spherical quantum well with infinitely high walls [7]. The oscillator strength, $f_{n,l}^{n,l}(a)$, between the the states (n_e,l_e) and (n_e,l_e+1) of an electron is described by the formula[9]:

$$f_{n,l}^{n,l}(a) \ = \ 4 \ (\Delta E_{n,l}^{n,l+1}(a)/E_e)\left[D_{n,l}^{n,l+1}(a) \ /e \ a_e\right]^2 \quad (5)$$

where E_e is the exciton binding energy:

$$E_e = (me/m_0)\varepsilon^{-2}Ry_0 \quad (6)$$

and Ry_0= 13.606 eV is the Rydberg constant.

The lifetime of the relaxation from the state (n_e,l_e) to (n_e,l_e+1) of an electron, can be described by the expression:

$$\tau_{n,l}^{n,l+1}(a) = (\frac{3\hbar}{\varepsilon E_e \, f_{n,l}^{n,l+1}(a)})(E_e/\Delta E_{n,l}^{n,l+1}(a))^2(c \ m_e \ a_e \ /\hbar)^3 \quad (7)$$

where c is the speed of light. Formula (6) gives for $E_e \approx$ 80 meV in the $CsPbBr_3$ perovskite NCs, $E_e \approx$ 48 meV for $FAPbBr_3$ and $E_e \approx$ 58.3 meV for the {en}$FAPbBr_3$ perovskite NCs.

Using Eqs. (2)-(7), we compared the lifetimes $\tau_{n,l}^{n,l+1}$ for the corresponding electron states in pure $FAPbBr_3$ NCs and doped {en}$FAPbBr_3$ perovskite NCs. For $FAPbBr_3$, the transition between the (n_e=1,l_e=0) and (n_e=1,l_e=1) electron states give a lifetime $\tau_{1,0}^{1,1} \approx$ 10 ns, while for the transition with (n_e=1,l_e=1) and (n_e=1,l_e=2) $\tau_{1,1}^{1,2} \approx$ 8 ns. A similar evaluation gives $\tau_{1,0}^{1,1} \approx$ 14 ns, $\tau_{1,1}^{1,2} \approx$ 10 ns, and $\tau_{1,2}^{1,3} \approx$ 8 ns for the case of {en}$FAPbBr_3$ NCs. The overall relaxation appears as a cascade transition process where the total effective lifetime of the relaxation after excitation is a convolution of the exponential decays for each step in the cascade. This results in a total effective lifetime which is the sum of each individual transition lifetime: ($\tau_{1,0}^{1,1}$ + $\tau_{1,1}^{1,2}$) $\approx$ 18 ns for $FAPbBr_3$ NCs and ($\tau_{1,0}^{1,1}$ + $\tau_{1,1}^{1,2}$ + $\tau_{1,2}^{1,3}$) $\approx$ 32 ns for {en}$FAPbBr_3$ NCs. This qualitatively explains that the deceleration of the PL emission is correlated with the absorption band-edge broadening observed in these perovskite NCs.

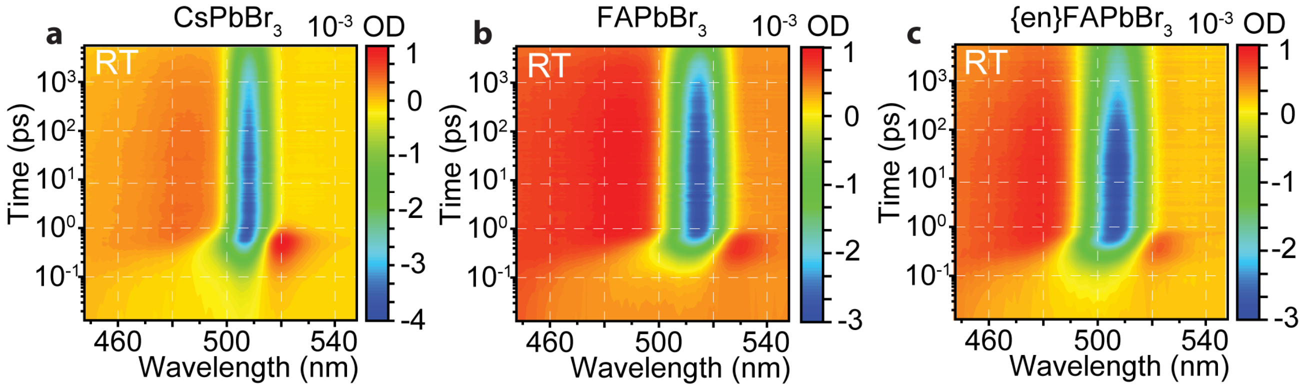

Supplementary Figure 3. 2D pseudo-colour plots for RT transient absorption of NCs colloidal solutions: (a) $CsPbBr_3$, (b) $FAPbBr_3$ and (c) {en}$FAPbBr_3$.

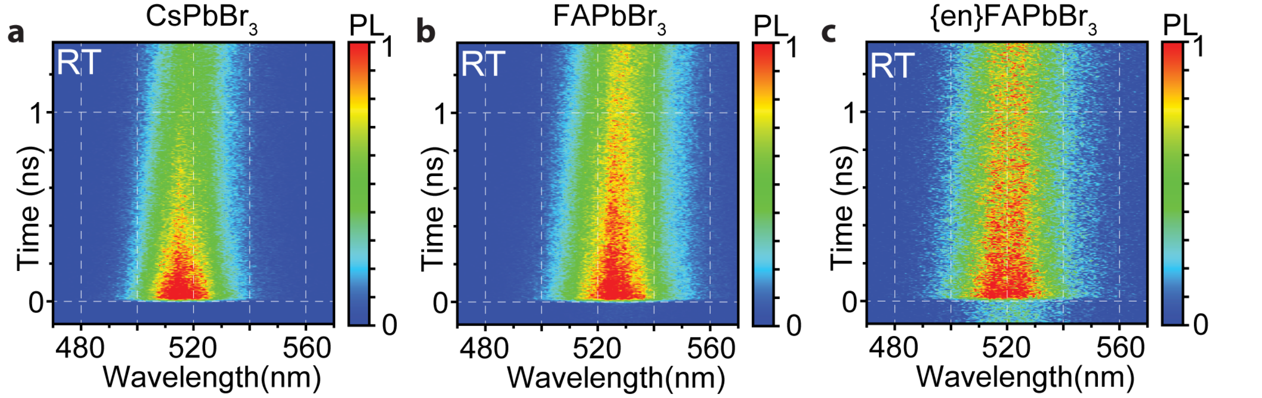

Supplementary Figure 4. 2D pseudo-colour plots for Streak-camera –recorded RT transient PL for NCs films: (a) $CsPbBr_3$, (b) $FAPbBr_3$ and (c) {en}$FAPbBr_3$.

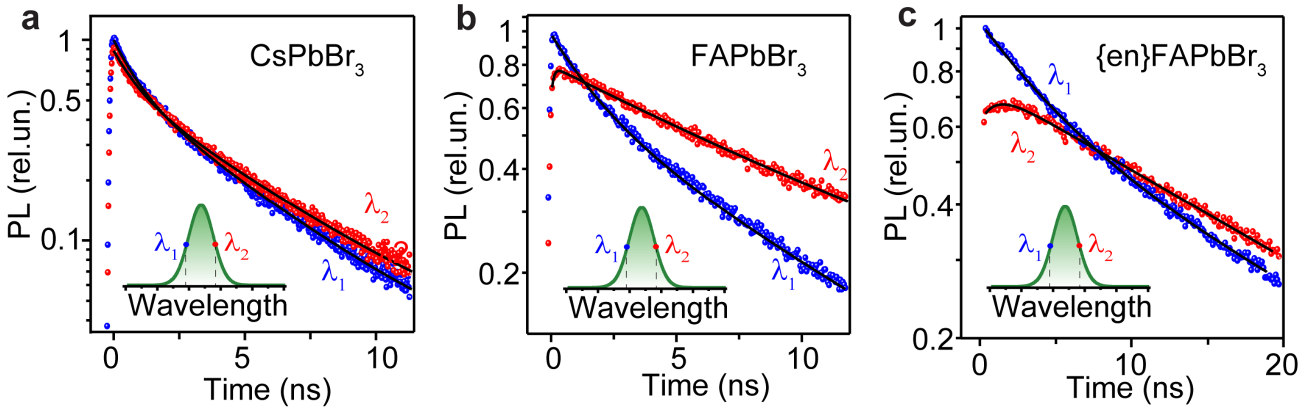

Supplementary Figure 5. Comparison of TCSPC-acquired time-resolved PL traces for NCs films recorded at RT from blue (λ1) and red (λ2) emission edges at 50% of maximal intensity (see inset sketches): (a) $CsPbBr_3$, (b) $FAPbBr_3$ and (c) {en}$FAPbBr_3$.

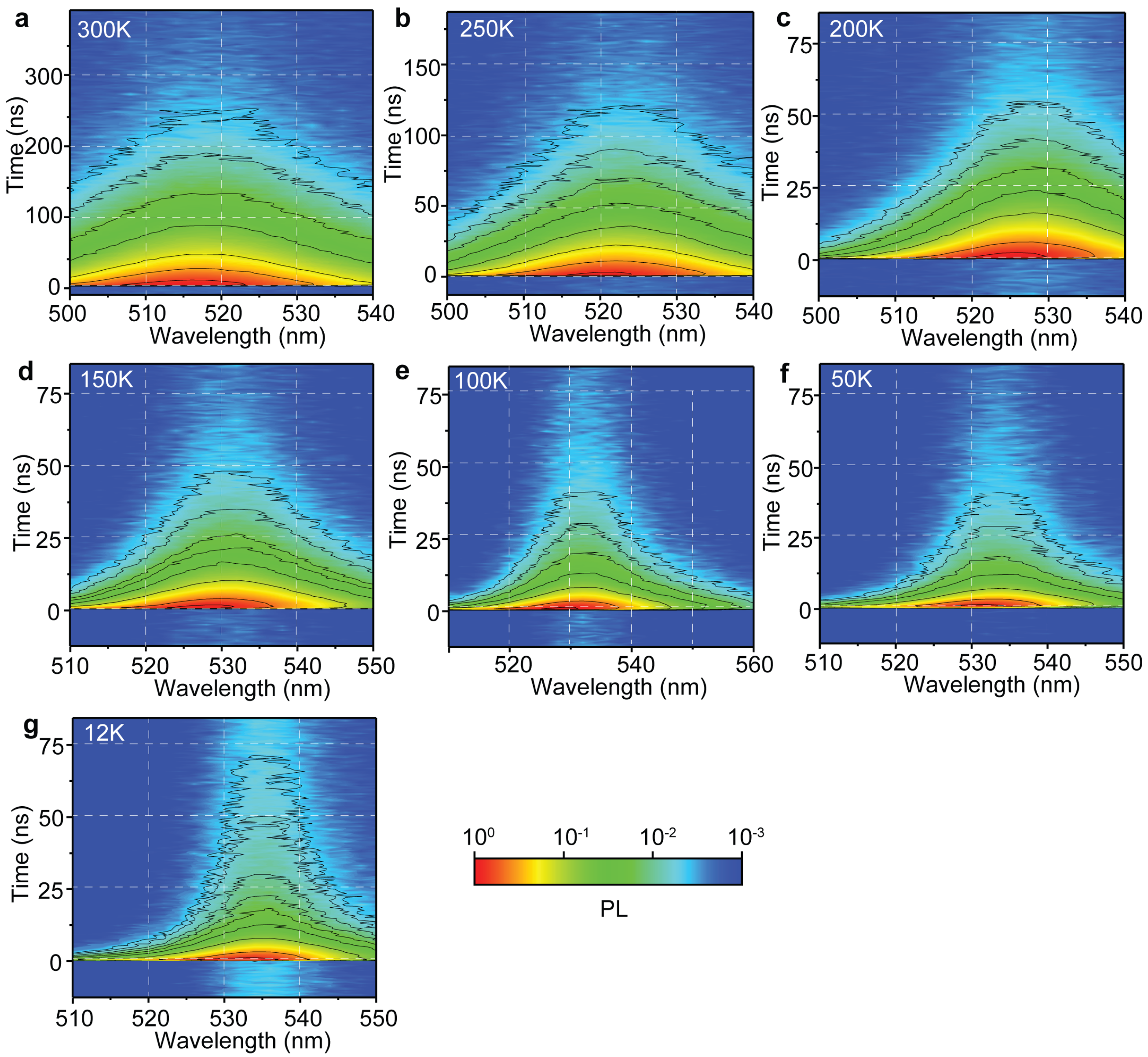

Supplementary Figure 6. (a)-(g): 2D pseudo-colour plots for temperature variation (12 K-300 K,) of TCSPC-acquired time-resolved emission spectra (TRES) for NCs {en}FAPbBr3 film.

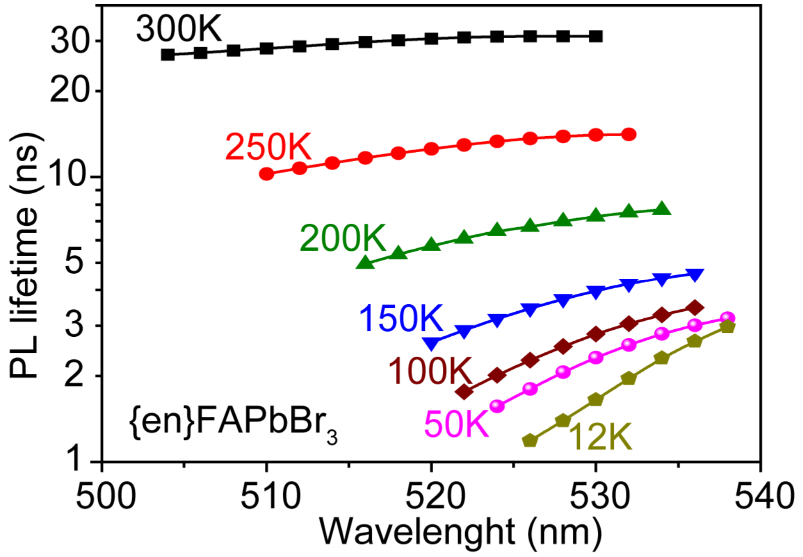

Supplementary Figure 7. Temperature variation (12 K-300 K) of a dependence (within central FWHM spectral range) for PL lifetime for NCs {en}FAPbBr3 film fitted from the data demonstrated on Supplementary Figure 6.

Supplementary Note 2. Advantages of EHD printing over traditional ink-jet printing.

Standard inkjet printing is not a suitable solution for the technology presented in this work; the minimal droplet volume achievable with standard inkjet printing is in the range of picoliters, meaning the printing resolution is typically limited to ~10 µm.10 Inkjet printing also suffers from coarse droplet placement precision, which would make the spatial co-alignment of the patterns of the two different inks very difficult or nearly impossible (see Supplementary Note 3 and Supplementary Movie 1 for EHD printing procedure, Supplementary Figure 8 for 2-ink pattern alignment).

Moreover, EHD printing was necessary to circumvent further wetting and vaporization problems associated with inkjet printing, such as coffee ring stains and non-uniform printed spots. In using EHD printing, we were able to precisely control the properties of the printed droplets of the two different inks, through optimization of the concentration of the ink, actuation voltage used, as well as nozzle size, in order to match the printed spots in size, uniformity and brightness, which was necessary to hide the QR code pattern.

It is a complex engineering problem to improve inkjet printing resolution and quality of printed spots. Reducing the inkjet printing nozzle size to obtain smaller droplets is challenging and costly

and would further require high printing actuation effort. Reducing the droplet size ejected by inkjet printing also worsens another problem of inkjet printing itself: droplet trajectory variation or full stop by air drag11.

Supplementary Note 3. EHD printing procedure.

To print one spot of the QR code image, AC voltages of 250-350 V_p at 1 kHz AC frequency are applied to the nozzle continuously, for a given pre-defined time length, which we denote as the total 'pulse' length. The use of an alternating current is necessary to minimize electrostatic charging effects on the non-conductive glass substrate. Each change in voltage polarity leads to a controllable deformation of the ink meniscus at the nozzle opening, and the ejection of attoliter-sized droplets, followed by an equal amount of oppositely charged droplets ejected after the polarity switches. The total pulse length and magnitude of the voltage applied are tuned according to the desired spot size and brightness. For each pulse length, several droplets land at the same location, with the solvent from each droplet evaporating before the arrival of the next, leaving behind only the nanoparticle content. In this manner, the nanoparticles from several consecutive droplets collectively form one brightly emitting spot at a well-defined position. The intensity of the emitted light by a given spot is directly proportional to the number of deposited quantum dots in that spot. Once the pulse length is completed, the stage moves to the next defined position and the ejection process is repeated. Thus, the substrate can be moved to create arbitrary patterns on demand.

Given that the two inks employed have different flow rates, we were identified the optimal printing parameters to yield the same printing results for each ink. Specifically, to achieve a unicolour perovskite printed image, the deposited spots from each ink have to be matched both in size and brightness/intensity of emitted light. To do so, a printing parametric test was conducted for each ink, prior to the final QR image printing. Multiple arrays of dots were printed with each ink, incrementally varying the ejection voltage and pulse length for each array.

The samples obtained were examined first with the in-situ confocal laser microscope of the printing setup, for visual inspection of the size of the printed dots, and then in a separate fluorescence microscope for a more complete assessment of the brightness of the dots.

Supplementary Note 4. Selection of 1,3,5-triethylbenzene as optimal solvent for EHD printing: Initially, printing feasibility tests were performed with toluene-based nanoparticle inks. Although toluene offers suitable nanoparticle dispersion stability, toluene-based inks are difficult to print. Unstable printing behavior was observed, such as uncontrollable spraying, and satellite droplet formation on the substrate. Moreover, the relatively high evaporation rate of toluene lead to fast clogging of the nozzle orifice, effectively terminating the printing process.

In fact, one of the most critical parameters for the ink formulation is the solvent's dielectric constant and evaporation rate. The latter needs to be low enough to avoid clogging at the nozzle (order of a micron nozzle opening) yet high enough to ensure fast evaporation once a droplet has landed on the substrate, in order to avoid puddle formation on the substrate and ensure high resolution patterning. After many rounds of optimizing the ink formulation for printing, we have found that 1,3,5-triethylbenzene fulfills these conditions quite well, and is also a suitable liquid for stable nanoparticle dispersions.

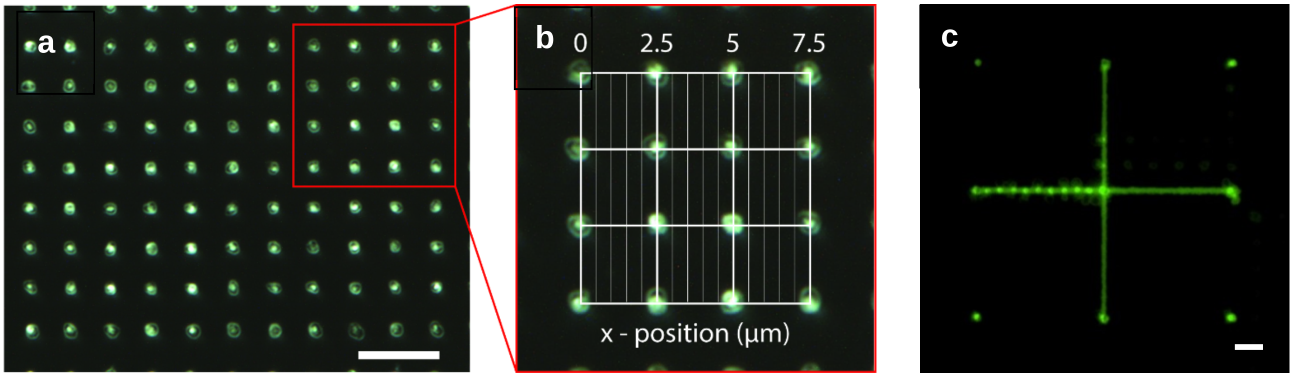

Supplementary Figure 8. EHD Printing and alignment accuracy. Fluorescence microscope images in dark field mode showing: (a) printed dot array using CsPbBr3 nanoparticle ink, (b) the zoomed-in image on the right features an overlaid grid with 500 nm intervals (scale bar is 5 µm), (c) the alignment scheme for the two nanoparticle inks (scale bar is 10 µm.). The cross-alignment marker is printed with the first ink, while the dots overlaying its lines are printed with the second ink.

Supplementary Note 5. Alignment procedure for EHD printing with two inks.

The printed QR code and its complementary image are each printed with different ink, and hence with two different capillary nozzle print-heads. Consequently, the second nozzle has to be carefully aligned with the first printed image. In the printing setup, the print-head is stationary while the stage holding the substrate is moved to create the desired printed pattern. Given that the mounted sample is left untouched on the stage for the entire duration of the image printing, there is no rotational misalignment. Before the printing of the first desired image, a cross marker is printed at a well-defined location, with a line along each axis. When switching to the second ink, the new nozzle is aligned to the existing marker, with a precision of less than 5 µm. Multiple points are then printed with the second ink, along the existing lines of the marker. Each point is offset from its predecessor in the direction normal to the printing direction by 100 nm. Using the high-resolution iSCAT microscope embedded in the printing setup, the printed dot with no offset to the marker line is determined. After establishing this information for each of the axes, the starting point of the printing software program is reset accordingly, and it is possible to achieve an alignment error of less than 100 nm.

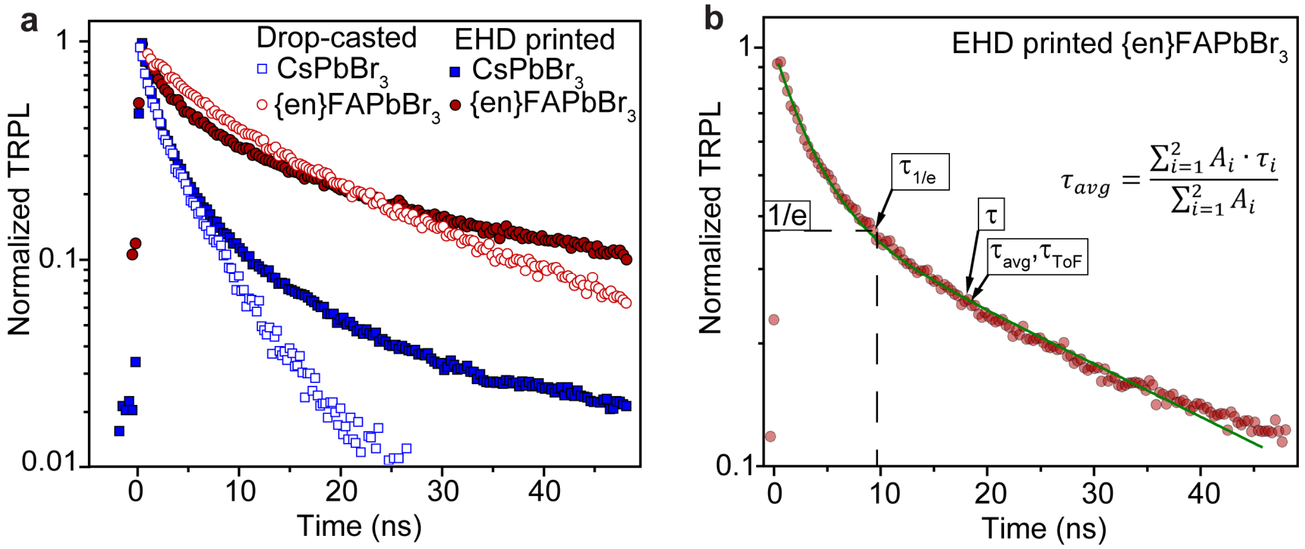

Supplementary Figure 9. Difference in lifetime estimation. (a) Comparison of TCSPC-acquired time-resolved PL traces for drop-casted and EHD printed $CsPbBr_3$ and $\{en\}FAPbBr_3$ films. (b) Illustration of the effect of the method of lifetime determination for non-monoexponential decay traces: a trace for an EHD printed $\{en\}FAPbBr_3$ film exhibits variation in estimated lifetime as depicted by the points at 1/e intensity ($\tau_{1/e}$ or so-called "fast-lifetime" used in TCSPC-FLIM setup), τ-lifetime parameter from rough single-exponential fit, and the value of the averaged lifetime: $\tau_{avg} = \frac{\sum_{i=1}^{2} A_i \cdot \tau_i}{\sum_{i=1}^{2} A_i}$ from fitting with a biexponential model, used in a single-point TCSPC-TRPL setup.

Supplementary Note 6. Difference between the evaluation of 1/e decay time (fast-lifetime) and averaged lifetime. The non-monoexponential behavior of TRPL traces (Supplementary Figure 9a) adds a certain complexity to the comparison of the PL lifetimes determined by different instruments. In the spectroscopic studies with relatively narrow data sets it is reasonable to carefully fit the individual TRPL traces and evaluate the so-called average lifetimes (amplitude-weighted, $\tau_{avg} = \frac{\sum_{i=1}^{2} A_i \cdot \tau_i}{\sum_{i=1}^{2} A_i}$) with a bi-exponential model, while in the FLIM experiment data sets are much larger and thus it is more time-effective and also more accurate to simply perform a "fast-lifetime" evaluation or in other words the delay time that it takes for a 1/e drop in the trace. The more that the decay behavior diverges from that of a monoexponential decay, the greater the difference will be in the lifetimes evaluated by the methods described above (see as example Supplementary Figure 9b).

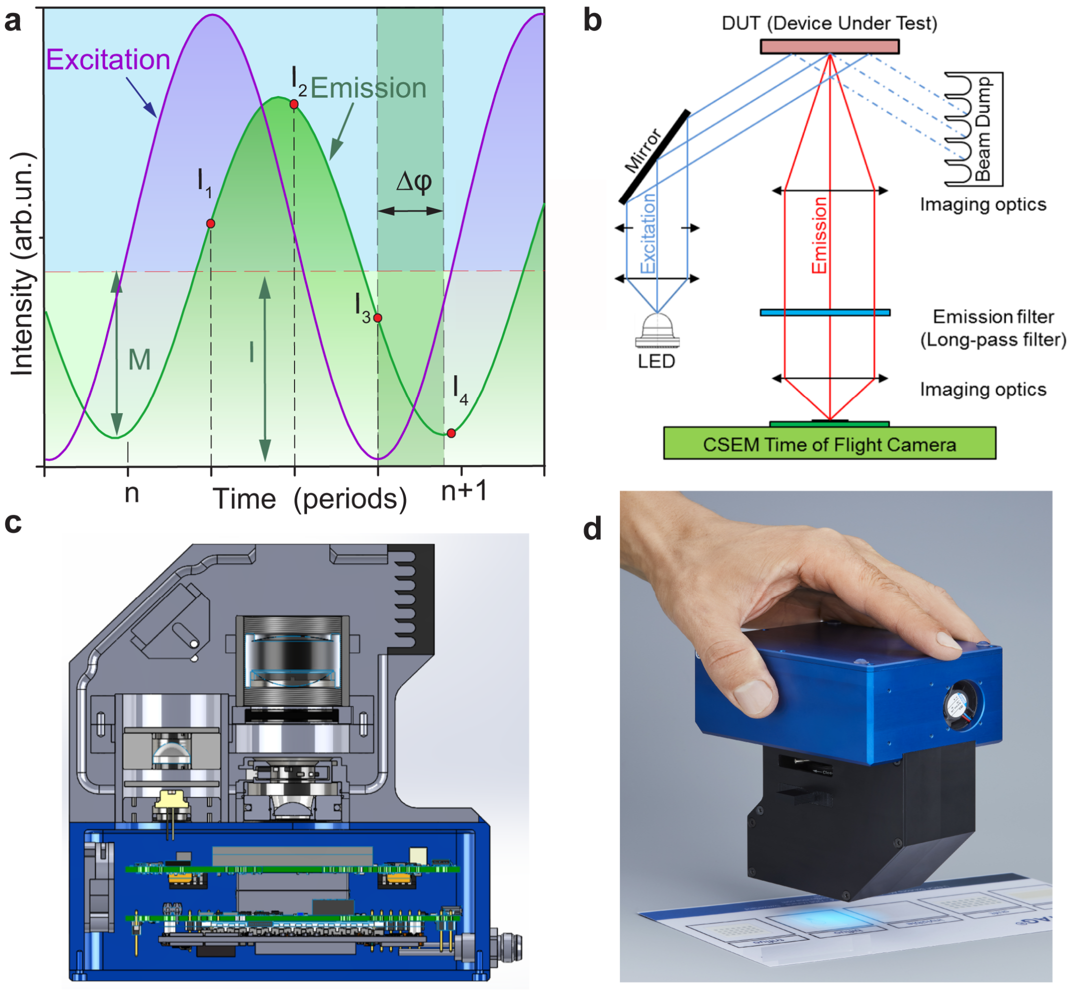

Supplementary Figure 10. ToF-FLI prototype. (a) Principle of recalculation of phase-locked intensities to obtain a phase-shift in the frequency domain for ToF-camera. (b) Optical scheme and (c) computer-aided design image of the ToF-FLI portable imager. (d) Example of use of ToF-FLI prototype for validation of lifetime-encoded image. Size of the prototype is about $10x10x10cm^3$.

Supplementary References

1 Protesescu, L. *et al.* Nanocrystals of cesium lead halide perovskites ($CsPbX_3$, X = Cl, Br, and I): novel optoelectronic materials showing bright emission with wide color gamut. *Nano Lett.* **15**, 3692-3696, doi:10.1021/nl5048779 (2015).

2 Yang, Z. *et al.* Impact of the halide cage on the electronic properties of fully inorganic cesium lead halide perovskites. *ACS Energy Lett.* **2**, 1621-1627, doi:10.1021/acsenergylett.7b00416 (2017).

3 Mannino, G. *et al.* Temperature-dependent optical band gap in $CsPbBr_3$, $MAPbBr_3$, and $FAPbBr_3$ single crystals. *J. Phys. Chem. Lett.* **11**, 2490-2496, doi:10.1021/acs.jpclett.0c00295 (2020).

4 Galkowski, K. *et al.* Determination of the exciton binding energy and effective masses for methylammonium and formamidinium lead tri-halide perovskite semiconductors. *Energy Environ. Sci.* **9**, 962-970, doi:10.1039/C5EE03435C (2016).

5 Liu, Y. *et al.* Investigation on binding energy and reduced effective mass of exciton in organic-inorganic hybrid lead perovskite films by a pure optical method. *Opt. Lett.* **44**, 3474-3477, doi:10.1364/OL.44.003474 (2019).

6 Pokutnyi, S., Gorbyk, P., Machno, S., Prokopenko, S. & Salejda, W. Excitons states in semiconductor quantum dots. *Int. J. Adv. Res. Phys. Sci.* **3**, 39-47 (2016).

7 Pokutnyi, S. Exciton states in quasi-zero-dimensional semiconductor nanosystems. *Semiconductors* **46**, 165–170, doi:10.1134/S1063782612020194 (2012).

8 Landau, L. D. & Lifshits, E. M. *Electrodynamics of continuous media*. (Pergamon Press, 1960).

9 Pokutnyi, S. I. Polarizability of germanium quantum dots with spatially separated electrons and holes. *Eur. Phys. J.* **135**, 74-83, doi:10.1140/epjp/s13360-019-00050-x (2020).

10 Derby, B. Inkjet Printing of Functional and Structural Materials: Fluid Property Requirements, Feature Stability, and Resolution. *Annual Review of Materials Research* **40**, 395-414, doi:10.1146/annurev-matsci-070909-104502 (2010).

11 Wang, Y., Bokor, J. & Lee, A. T. in *SPIE Advanced Lithography.*
