# Supplementary material for: Radiative lifetime-encoded unicolour security tags using perovskite nanocrystals
Source: Nat Commun. 2021 Feb 12;12:981. doi: 10.1038/s41467-021-21214-3 (PMC7881120; doi:10.1038/s41467-021-21214-3)
Supplement: Supplementary file 2 — Description of Additional Supplementary Files [file 41467_2021_21214_MOESM2_ESM.pdf]

Description of Additional Supplementary Files

File Name: Supplementary Movie 1
Description: **EHD printing of QR code pattern using $CsPbBr_3$ ink.** Video of the microscope-attached camera with an observation of the EHD printing procedure of QR code with $CsPbBr_3$ ink.
